# Beyond Time Saved: Implementation, Equity, and the Utility Threshold for Nursing AI Scribes

**DOI:** 10.2196/101190

**Published:** 2026-05-27

**Authors:** Charlene E Ronquillo

**Affiliations:** 1 School of Nursing, Faculty of Health and Social Development, University of British Columbia, Okanagan Campus, 1147 Research Road, Kelowna, BC, V1V1V7, Canada, 1 250-807-8180,

**Keywords:** artificial intelligence, long-term care, nursing informatics, speech recognition, health equity, digital health

## Abstract

Schwabe et al’s pre-post time-motion study of a domain-specific artificial intelligence (AI) speech assistant used by nurses in German long-term care provides one of the few real-world, full-shift evaluations of an AI scribe deployed to a nonphysician workforce, with paired objective observation and self-reported outcomes. This commentary points to the implications of these findings that extend well beyond the time savings headline. The study reports substantial reduction in self-reported documentation time and increased satisfaction with the documentation system, yet workplace satisfaction and the perception that AI scribes are “a good idea to implement” did not improve. Taken together, these findings show three undertheorized issues for AI scribe implementation in nursing and long-term care. First, postimplementation increases in time spent reviewing entries and retrieving information indicate that AI scribes redistribute cognitive effort from authoring to verification, with unknown consequences for satisfaction, mastery, and error detection. Second, the apparent paradox of rising documentation satisfaction alongside falling expectations of AI quality represents user calibration. Third, the substantial equity considerations of automatic speech recognition documentation reflect a broader trend of AI scribe studies that treat equity as a caveat, rather than treating equitable performance as empirically measurable and testable across variations in linguistic styles, dialects, and social linguistic dimensions. To advance the field, the next generation of nursing AI scribe research must treat documentation as a heterogeneous bundle of authoring, reviewing, retrieving, and verifying activities with distinct satisfaction and error profiles; specify and validate end-user–defined anchor utilities, rather than having a narrow focus on diffuse improvement; and treat equity testing and reporting of both automatic speech recognition systems and workforce adoption as standard reporting expectations, rather than caveats.

Schwabe et al’s [1] study is among the few carefully designed real-world evaluations of an automatic speech recognition (ASR) artificial intelligence (AI) scribe in nursing to date, a domain substantially underevaluated relative to physician documentation [2]. The full-shift, pre-post time-motion design and the choice of a domain-specific speech recognition system trained on nursing language are methodological advances over the small physician-focused pilots that dominate the AI scribe literature [2]. Like much of that literature, Schwabe et al [1] is a vendor-conducted evaluation: the first author and all observers were GmbH employees, developers of the ASR AI scribe used in the study. The methodological advance credited here lies in evaluation setting, duration, and analytic transparency, not independence from the vendor. In this commentary, attention is pointed to three findings that generate research questions the field has yet to substantively engage: (1) documentation work is reshaped, not only reduced; (2) the paradox of rising satisfaction alongside falling quality expectations can be interpreted as a utility threshold; and (3) the equity considerations of ASR-based documentation are treated as a caveat rather than empirically tested.

## Documentation Work Is Reshaped, Not Only Reduced

The reduction in long-term care nurses’ self-reported documentation time (Δ=−31.14 min) is accompanied by an acknowledged but less-discussed compositional change from authoring toward verification and information retrieval: time spent reviewing entries rose from 0.82 to 1.47 minutes, and information retrieval rose from 3.48 to 4.85 minutes [1]. The authors note the inability to directly link reduced documentation time to changes in documentation quality. This surfaces an open empirical question that the existing AI scribe literature has rarely disaggregated [2]: whether the review and verification of ASR-produced documentation produces the same satisfaction, mastery, and confidence as clinician authoring, or whether it changes error detection, omission, or copy-forward propagation. The next generation of evaluation should treat documentation as a heterogeneous bundle of authoring, reviewing, retrieving, and verifying activities, each potentially carrying distinct satisfaction and error profiles.

## The Utility Threshold and the “Good Idea” Paradox

Nurses’ expectations that AI scribe use would improve documentation quality were lower post test than at baseline, even as satisfaction with the documentation system itself rose, and perceptions that it was a “good idea to implement” slightly decreased [1]. Building on technology acceptance literature [3], this pattern can be interpreted as signaling calibration rather than disappointment, from which the construct of a *utility threshold* emerges: the minimum demonstrable day-to-day clinical value an AI scribe must produce for nurses to endorse its sustained use. Perhaps counter to developer perceptions, the utility threshold may not be for an AI scribe to “do everything well” but “do at least one thing well enough that I notice it in my shift.” The reduction in overall documentation time per shift may indeed be sufficient for clinicians to continue using it, even where documentation quality is not markedly improved. The findings on reduced daily interruptions deserve separate emphasis: interruptions to nursing work are associated with loss of concentration and focus, delays in planned tasks, and incomplete work, and are major contributors to medication errors [4,5]. Any tool that meaningfully reduces interruptions warrants a dedicated patient safety study. Finally, the privacy and confidentiality work that nurses absorb when dictating at the bedside, in corridors, and in shared spaces was not addressed in the study and offers one explanation for why “good idea” perceptions did not rise alongside other satisfaction measures. AI scribe design should validate explicitly identified anchor utilities such as overall time savings, reduced interruptions, privacy trade-offs, or yet-to-be-identified gains, rather than diffuse improvement. The greatest promise of clinical benefit will arguably stem from these anchor utilities being defined by end users.

## Equity as an Underexamined Dimension

The equity stakes of ASR-based documentation are substantial but underexplored. In a US home health care setting, an evaluation of four ASR systems (two commercial: AWS General, AWS Medical; two open-source: Whisper, Wav2Vec 2.0) against gold-standard transcriptions of patient-nurse encounters found that all systems performed significantly worse for Black patients than White patients, with the largest discrepancies in affective and social linguistic dimensions, particularly relevant for nursing assessment of social cues [6]. The mechanism is structural. Training data underrepresent dialect and register variation [6], resulting in a documented pattern across major commercial ASR systems with differences in average word error rates among speakers of different racial backgrounds [7]. It is useful to distinguish speaker-level ASR bias (dialect, accent, vocal aging) from system-level training bias (sampled at training): speaker-level bias can sometimes be mitigated through fine-tuning, whereas system-level bias requires upstream intervention beyond downstream implementers’ reach. Acknowledgment by Schwabe et al [1] of differential performance across dialects, accents, and terminology, while a good start, illustrates a pattern among AI scribe studies that treat equity as a caveat rather than an empirically testable and measurable dimension of technical performance. For example, the German nursing workforce in the study [1] includes regional dialect, generational, and language-of-origin variation that can serve as equitable ASR performance axes for empirical testing and measurement. A second equity dimension lies at the intersection of workforce characteristics and AI scribe adoption. The authors acknowledge that continued tool engagement may differ by career stage, training status, and role-related workload [1], and can extend to age, language-of-origin, prior digital health experience, and other established predictors of technology acceptance. Addressing the ASR equity gap rather than treating it as an afterthought requires conducting subgroup analyses by context-specific axes of linguistic variation, including accent, dialect, and professional characteristics as suggested [1], and disaggregated reporting on adoption, dropout, and equity metrics of ASR performance.

## A Research Agenda

Table 1 summarizes research priorities following each thread. The Schwabe et al [1] study, despite limitations, contributes what hopefully becomes a precedent for stronger designs and longer time horizons of AI scribe and nursing AI studies. While the 8-week pilot is relatively brief, it offers important insight into implementation factors that determine sustained use, including workflow fit, staffing stability, and organizational readiness [8]. The time-motion methodology and pairing of objective and self-reported outcomes are the kind of evidence nursing AI scribe research needs more of. The implementation, equity, and utility-specification work, however, remains.

**Table 1.** Schwabe et al [1] findings, commentary interpretation, and research priorities for nursing artificial intelligence scribe research.

Schwabe et al [1] finding	Commentary interpretation	Research priority
↓ Self-reported documentation time (Δ=–31.14, SE 6.57 min)	Time savings real; sustainability beyond 8 weeks unknown	Longitudinal (≥12 mo) implementation studies anchored in implementation science framework (eg, CFIR^a^ inner-setting constructs [8,9])
↑ Reviewing entries (0.82 → 1.47 min); ↑ information retrieval (3.48 → 4.85 min)	Clinical documentation task composition shifts from authoring to review and verification	Disaggregate documentation as a heterogeneous bundle of activities; examine satisfaction, mastery, and error implications of each
↑ Documentation satisfaction; ↓ quality expectations; “good idea” not improved	Utility threshold reached; unaddressed privacy and confidentiality labor a possible explanation for stagnant “good idea” perceptions	Specify and validate end-user–defined anchor utilities; investigate privacy and confidentiality protocols for bedside dictation
↓ Daily interruptions	Patient safety relevance, not workflow nicety [4,5]	Test whether interruption reductions translate into measurable reductions in nursing error, omission, or delay
Workplace satisfaction and “good idea to implement” unchanged	Adoption gains ≠ workforce benefit; possible utility threshold not yet reached for sustained use	Mechanism studies of what drives sustained use vs initial adoption
ASR^b^ equity not empirically tested	Bias likely along regional, generational, language-of-origin axes [6,7]	Equity audits as standard reporting: ASR performance disaggregated by context- and domain-relevant patient/speaker characteristics; adoption disaggregated by workforce demographic

a CFIR: Consolidated Framework for Implementation Research.

b ASR: automatic speech recognition.

## Abbreviations

AI: artificial intelligence
ASR: automatic speech recognition
